# Integrative Neuro-Endocrine Pathways in the Control of Reproduction in Lamprey: A Brief Review

**DOI:** 10.3389/fendo.2013.00151

**Published:** 2013-10-18

**Authors:** Mihael Freamat, Stacia A. Sower

**Affiliations:** ^1^Department of Molecular, Cellular and Biomedical Sciences, Biochemistry Program, Center for Molecular and Comparative Endocrinology, University of New Hampshire, Durham, NH, USA

**Keywords:** lampreys, behavior, GnRH, GnRH receptor, sex steroids, pheromones, photoperiod

## Abstract

The gonadotropin-releasing hormone (GnRH) system is well known as the main regulator of reproductive physiology in vertebrates. It is also part of a network of brain structures and pathways that integrate information from the internal and external milieu and coordinate the adaptive behavioral and physiological responses to social and reproductive survival needs. In this paper we review the state of knowledge of the GnRH system in relation to the behavior, external, and internal factors that control reproduction in one of the oldest lineage of vertebrates, the lampreys.

## Introduction

Neuro-endocrine integration is defined as the process whereby a neuro-endocrine system converts signals received from the external and internal media into adaptive physiological and/or behavioral changes (1). Reproduction in all vertebrates is controlled by neuro-endocrine mechanisms that adjust endocrine status and behavioral output to internal (hormonal and metabolic) milieu and to external environmental and social conditions (2, 3). Hypophysiotropic gonadotropin-releasing hormone (GnRH) peptides induce changes in gonadal physiology via the hypothalamic-pituitary-gonadal (HPG) axis. At the same time, they represent the secretomotor output of a neuro-endocrine integrative pathway that involves multiple interconnected brain structures (4). These structures are responsive to the reproductive status of the animal primarily via the circulating gonadal steroid levels. In addition, they receive and process exteroceptive input from the environment directly or indirectly through different sensory modalities. The structure and dynamics of this system may differ in different lineages of vertebrate animals likely as a result of adaptive changes connected to their life cycle, ecological niche, and degree of elaboration of their central nervous system. The overall layout of this regulatory system however appears to be remarkably conserved, likely reflecting an ancestral condition that evolved long before the divergence of Gnathostome lineages.

Lampreys are jawless aquatic animals represented by a number of species, most of them in the northern hemisphere but two species (*Geotria* and *Mordacia*) can be found in the southern hemisphere as well. Lampreys (*Petromyzontidae*) and hagfishes (*Mixini*) are the only members of the monophyletic vertebrate group of Cyclostomes and the only agnathans surviving in the actual fauna. The overall body morphology of lampreys seems to have been surprisingly well conserved over the 400–500 millions years of evolution as suggested by paleontological evidence (5, 6). Although small, their brain shows the typical morphological organization of vertebrate brain and most of the main neuroanatomical structures in Gnathostomes can be homologized with lamprey brain (7). Lampreys belong to the Group I of vertebrate taxa, characterized by limited neuronal proliferation and migration. In contrast, the hagfish is considered a member of the Group II of vertebrates, with a considerable independent divergent elaboration of its brain (8). Therefore, the lamprey brain might be the most representative for the last common agnathan ancestor of all vertebrates (7, 9).

Lampreys have a simple reproductive cycle yet many of the vertebrate behavioral patterns of reproduction are present, including appetitive and consummatory components, mate choice, and aggression. Although not the most convenient animal model, it is much easier to study compared to the hagfish, a deep-sea animal for which many aspects of its reproduction remain a mystery even after a century of study. The lampreys are particularly convenient as a model organism for the development of the brain and reproductive system as its larvae can be found burrowed in the mud of many of the European and North American streams. In addition, there is a significant economic interest in lamprey reproduction driven by the search for methods of control of the invasive landlocked sea lamprey population in the Great Lakes (10).

We begin with a brief overview of the main events of the reproductive behavior of lampreys. We follow this with an overview of the current knowledge available on the GnRH system of lamprey and of the role of lamprey GnRH in its reproductive biology. The next section is dedicated to the internal signals that convey information relevant for reproduction, in particular the sex steroids known to date in lamprey and their central feedback. The last two parts are a brief survey of the current information regarding the external, environmental, or social (pheromonal) factors known to influence lamprey reproductive behavior and physiology.

## Lamprey Reproductive Behavior

The most studied species in the North America are the sea lamprey (*Petromyzon marinus*) and the Pacific lamprey (*Entosphenus tridentatus*). Sea lamprey is represented by two varieties: the landlocked lampreys in the Great Lakes and the anadromous variety in the Atlantic Ocean seaboard. Due to the extraordinary measures that were taken for the control of the lamprey population in the Great Lakes (10), the biology of the landlocked form is relatively better understood compared with the Atlantic anadromous form or with the Pacific lamprey, particularly in respect to the role of pheromonal communication and populational estimates. Although some differences exist, the biology of the predatory lamprey species is similar and the results obtained in one species can usually be extrapolated to other.

Lamprey reproduction follows a linear progression. Reproductive steroidogenesis and gametogenesis occur as a single event that culminates during the spawning season. Phases of the lamprey life cycle are (11): (1) larval stage: benthic larvae burrowed in the soft mud of the ocean or lake tributaries, filter feeding. Length of this stage differs wildly even within the same species of lamprey and in the same geographical area. (2) Metamorphosis from larvae to adult. (3) Macrophtalmia or juvenile lampreys: during this phase the animals become parasitic and start feeding. Juveniles migrate to their final feeding ground in the ocean (for anadromous forms) or lakes. (4) Parasitic phase. (5) Adult lampreys: the final stage in lamprey life cycle, reproductive mature individuals migrate upstream, spawn, and die.

Sexual maturation (spermatogonia proliferation, development of spermatocytes in males, and vitellogenesis in females) in lampreys is initiated during the parasitic phase of its life cycle. Final maturation takes place during upstream migration. Three main stages were identified for the upstream migration of reproductively mature lamprey: (i) initial migration from the ocean or lake to coastal rivers usually during the winter that precedes spawning, (ii) pre-spawning animals swim upstream the river, and (iii) spawning stage when sexual organs are fully matured and which includes specific reproductive behaviors like nest building, nest fanning, inter-male aggression, and the spawning act itself.

Among the environmental factors that influence the upstream migration and their behavior during the final spawning phase the most important are photoperiod and temperature. Hydrological factors may also impact migration (12, 13) although they are likely of lesser neurobiological relevance. Light and water temperature act in concert in determining the migratory activity of the animals. Lampreys are photophobic, avoid the daylight and only advance their migration at night. Migration and sexual maturation of adult lampreys takes place only within a specific range of water temperatures. The minimal and maximum temperatures of this range varies with the species and habitat, with the anadromous sea lampreys having a tendency to prefer a narrower domain of temperatures, between 15 and 20°C while the landlocked and Pacific lampreys being still active at water temperatures as low as 5°C. Optimal spawning temperature varies within 14–21°C in all lampreys species (14). These values also depend on the latitude, reflecting similar patterns in migration temperature preferences. Pattern of activity of the lamprey relative to photoperiod reverses during the last (spawning) phase of lamprey reproduction, animals engage in typical reproductive behaviors during the daylight (15).

Selection of the river stream by reproductively mature lampreys seems to be dependent primarily on chemical signals received from population of larvae residing in these rivers (10, 16). This pheromonal communication between different stages of the life cycle of lamprey was mostly studied in the landlocked form of *P. marinus*. The relative genetic homogeneity of the anadromous Atlantic sea lamprey on the North American east coast also suggests that mature animals do not return to their home streams for spawning (17).

Body size decreases during final migration by up 24% in the anadromous sea lamprey a value intermediary between Pacific lamprey (up to 30%) and landlocked sea lamprey (up to 10%) (15). This weight loss is associated with metabolic depression and organ deterioration, particularly of the digestive system. It is not known how these changes may impact the internal medium and influence the neuro-endocrine mechanisms associated with reproductive maturation and behavior. Toward the end of their upstream migration animals become almost blind (18) and probably rely mostly on olfactory stimuli to find their mate.

## Lamprey GnRH System

The canonical function of the vertebrate GnRH is to stimulate gonadal gametogenesis and steroidogenesis via the HPG axis. Molecular phylogenetic analysis of GnRH transcripts described to date suggests existence of three main types of vertebrate GnRH isoforms. Type 1 (formerly known as “mammalian” GnRH) is present in a majority of species with the exception of some fish. The type 2 GnRH (or “chicken” GnRH) shows the widest pattern of distribution, an identical GnRH type 2 was found in all major Gnathostome lineages. Type 3 is characteristic to teleosts (19). Most species studied to date have at least two isoforms, with distinct localization and specialized functions. Relationships between paralogous group and biological function of GnRH isoforms are not very strict. For example the GnRH 1 is missing in some fish species where the hypophysiotropic function is accomplished by GnRH 3. Gnathostome GnRH type 1 is found primarily in neurons of the preoptic nucleus of the hypothalamus that project to the ventral hypothalamus. High expression of GnRH type 2 is mostly found in the midbrain tegmentum with GnRH fibers sent primarily to other areas in the midbrain and in the hindbrain. The teleost GnRH type 3 is expressed in telencephalon in the ganglion of the *nervus terminalis* (20).

Therefore, in a very simplified GnRH type on biological function mapping scheme GnRH 1 is the hypophysiotropic form, GnRH 2 is considered mostly a neuromodulatory peptide with role in behavior while the teleost GnRH 3 could play various roles as mentioned, many times being found in the telencephalic area where some evidence suggests that it plays an olfactory modulatory role. The GnRH type 2 peptides are very well conserved in all vertebrates species with the exception of some of the mammalian groups where this peptide is absent, as it is the case of rodents and some primates. Behavioral control function hypothesized to be played by midbrain GnRH 2 seems to be supplanted by other systems in these species. Interestingly, in monkeys as well as in humans both GnRH type 1 and GnRH type 2 are present and show hypothalamic distribution albeit in distinct neuronal populations. This and the evidence collected mainly in monkeys on their hypophysiotropic role and differential estrogen neuronal sensitivity suggests that both isoforms are components of the HPG axis in these species with functions tuned to respond to different physiological contexts (21). The main point in respect to the function-phylogeny relationship for GnRH molecules is that all isoforms are expressed in neurons of the central nervous system of Gnathostomes locations and under neuromodulatory interactions that suggest their role in various mechanisms of reproductive physiology and behavior.

In lamprey three members of the GnRH family of neuropeptides have been identified: lamprey GnRH-I (22), lamprey GnRH-III (23), and more recently lamprey GnRH-II (24). In the lamprey GnRH decapeptides residues 1, 2, 4, 9, and 10 (pGlu, His, Ser, Pro, and GlyNH_2_ respectively) are common with the Gnathostome GnRH. Tyr^3^ and Leu^5^ in lGnRH-I, Asp^6^ in lGnRH-III, and Glu^6^ in lGnRH-I as well as Phe or Lys in position 8 are characteristic to lamprey sequences (24). Phylogenetic analysis of Chordate and Protochordate GnRH isoform transcript sequences strongly supports the hypothesis of a common ancestor for agnathan and Gnathostome GnRH. The precise relationship between lamprey peptides and the three subtypes of GnRH in the rest of the Vertebrates on the other hand was more difficult to resolve. Given their removed position in the molecular phylogenetic tree of the GnRH transcripts lGnRH-I and -III were initially classified as a fourth group of vertebrate GnRH peptides. More recent approaches based on synteny analysis of GnRH paralogs suggest that GnRH-I and -III belong likely to the type 3 GnRH group (25). The newest member of GnRH family in lamprey, lGnRH-II shows the highest similarity with Gnathostome sequences and it was suggested to be a direct descendant of an ancient form of type 2 GnRH (24, 25).

In contrast to Gnathostomes, the distribution of the three identified GnRH forms (-I, -II, and -III) in lamprey brains is much more restricted. Lamprey GnRH immunoreactive nerve fibers originate from cells in the arc-shaped hypothalamic/preoptic areas and end at the neurohypophysis forming the preoptico-hypophyseal GnRH tract (24, 26). The same pattern restricted to the preoptic/hypothalamic area was found by *in situ* experiments for lGnRH-I and -III transcripts (27). Presence of GnRH-II transcripts in the olfactory bulb and in the hindbrain in addition to hypothalamus suggested by *in situ* experiments were not confirmed by immunohistochemistry (24). This is similar to earlier reported immunohistochemical studies were both immunoreactive lamprey GnRH-I and -III were found in the cell bodies of the rostral hypothalamus and preoptic area in larval and adult sea lamprey and not expressed in extra-hypothalamic regions of the brain (28, 29).

Gonadotropin-releasing hormone receptors are G-protein coupled receptors acting primarily through interaction with Gq/11 G-proteins followed by activation of the IP3 second messenger signal transduction pathway (30). Activation of the cAMP dependent signaling pathway has been reported in some cases as well. Initially these receptors were classified in two groups: type I (receptors without an intracellular tail, typified by mammalian GnRH receptor I isoform) and type II (receptors with an intracellular tail, with a wider phylogenetic distribution). Absence of the cytoplasmic domain in a GnRH receptor was shown to have important consequences on the properties of the receptor in respect to signal transduction and receptor activity regulation (31). An approach more interested in the evolutionary significance of the multiple GnRH receptor isoforms identified three distinct classes of GnRH-Rs: type I (mammalian and non-mammalian tail-less receptors), type II, and type III GnRH-Rs. Vertebrate type I GnRH-Rs are represented in teleosts, amphibians, reptiles, and avian species and in mammals. The type II GnRH-Rs include receptors from amphibians, reptiles, and some mammals. Type III GnRH-Rs include sequences from teleost fish, amphibians, reptiles, and avian species but do not occur in mammals (32). When multiple isoforms of GnRH receptors are present in a species they tend to have low ligand specificity and wide pattern of expression both in the brain as well as in other tissues. In this respect it can be said that the GnRH/GnRH receptor pairs show a lower degree of co-evolution compared with other ligand receptor systems and specific functions of some of GnRH–GnRH receptor interacting partners may be controlled primarily at the level of regulation of their expression in specific locations (20).

The first evidence for existence of specific agnathan GnRH receptors was reported in 1994 by Knox et al. in anadromous sea lamprey. *In vitro* autoradiography followed by Scatchard analysis of binding of a mammalian GnRH analog to lamprey pituitary identified two classes of high affinity binding of the ligand. These binding sites were located in the *proximal pars distalis* and in a lesser extend in the *rostral pars distalis* of the pituitary. The labeled analog was displaced by the lamprey GnRH peptides I and III which suggested that the detected high affinity binding was due to the presence of at least two types of GnRH receptors expressed in the pituitary tissue of lamprey (33). Moreover, intensity of binding depended on the reproductive status of the animal, with a peak during the ovulation time in females (34). These results were confirmed in 2006 when the molecular cloning of the first lamprey GnRH receptor (lGnRH-R 1) was reported (35) and more recently in 2012 when the sequences and properties of two more lamprey receptors (lGnRH-R 2 and 3) were published (36). All lamprey receptors have an intracellular tail and based on their sequence similarity it has been proposed that they are orthologs of Gnathostome type II receptors (lGnRH-R 1) or of type III receptors (lGnRH-R 2 and 3) (32). All of them were shown to activate the IP3 accumulation in COS-7 cells. Only lamprey GnRH-R I was determined to induce the cAMP accumulation in transiently transfected COS-7 cells. Lamprey GnRH receptors are fairly promiscuous in respect to binding and activation by lamprey GnRH isoforms: all of them are activated in various extents by lGnRH-II and -III. The only specificity of interaction was found for lGnRH-I which was only capable of stimulating signal transduction via the lGnRH-R 1 (24, 35). The distribution of expression of lamprey GnRH receptors as reported from RT-PCR and *in situ* experiments (36, 37) includes the pituitary for all isoforms but also a much wider diversity of brain areas, including neuronal populations in the telencephalon, epithalamus, thalamus, preoptic area, and in the midbrain. Some sexual dimorphic features of this distribution was also noted (37).

In summary, the body of evidence accumulated on lamprey GnRH system strongly suggests its central role as a central regulator of lamprey reproductive physiology, similar to Gnathostomes. In contrast with the later evolved vertebrates however, the expression of all lamprey GnRH isoforms seems to be confined to the hypothalamus, particularly in its rostral, preoptic area. In mammals it has been shown that GnRH neurons are synaptically connected with tens of other functionally diverse brain areas (4) and therefore integrated into an extensive regulatory neural network. In lamprey little is known about the connectivity of GnRH neurons at this time other than their projections to the neurohypophysis.

## Gonadal Steroid Hormones in Lamprey

Of the five major classes of steroid hormones in vertebrates, i.e., the estrogens, progestagens, androgens, glucocorticoids, and mineralocorticoids, the first three are the products of the steroidogenic activity in the gonads and their activity is tightly correlated with the development and maintenance of male and female sexual characteristics during the embryonic and adult life. After their biosynthesis from cholesterol, they act in a paracrine fashion at gonadal level thus contributing to gametogenesis. They are also released in the general circulation, transported to various tissues where they regulate the development of the secondary sex characteristics. Sex steroids transported into the brain act as neuromodulatory chemicals on neuronal populations located in diverse areas of the brain. These neurons and their connections are an instance of the interoceptive circuits that relay information about changes in the internal states of the organism reflecting survival needs related to reproduction (38). Their effect depends on the relative ratios between estrogens, androgens, and progestagens, on the level of expression of their receptors and on sexually dimorphic structural features of the vertebrate brains (39). “Classical” sex steroid hormones, i.e., estradiol, testosterone, and progesterone are present in all classes of Gnathostomes along with orthologs of their specific nuclear receptors. Nuclear receptors for steroid hormones were one of the first transcription factors discovered (40). It has been long time recognized that in addition to the slow, long-term action the steroid hormones exert also faster, short term effects. Direct steroid interaction with nuclear steroid receptors is considered responsible for the slow effect of the hormones. The need to explain the mechanism by which estrogens, progestins, and androgens induce their fast response has induced a considerable interest in the study of the membrane action of steroid hormones and in their non-genomic effects (40). Steroid receptors are cytoplasm-nucleus shuttle proteins, their interactions with steroids induce complex non-genomic and genomic effects within the cell, trigger activation of other signaling pathways in addition to their nuclear import and direct activation of transcription. Their association with membrane can, in some circumstances activate other, membrane bound, receptors (41, 42).

Gonadal steroid estradiol and progesterone changes correlated with the reproductive stages were demonstrated in the Atlantic hagfish (43, 44). In Protochordates, no gonadal steroid hormones have been found in Tunicates. Screening of the *Ciona* genome has revealed neither steroid receptors nor the enzymes required for synthesis of sex steroids (45, 46).

Two putative sex steroids, estrone and progesterone were identified in the gonads of the Cephalochordate amphioxus. A complement of steroidogenic enzymes and an estrogen receptor (ER) and a steroid receptor orthologs were demonstrated as well. Regulatory changes in the expression of these genes during spawning suggest that these steroid hormones might play a role in gonadal maturation and reproductive function in this animal. However, the existence of a central neuro-endocrine control mechanism in Cephalochordates is still in the stage of unconfirmed hypothesis as no GnRH or other gonadal steroidogenic factor originating in their central nervous system was found (47). Treatment with synthetic octopus GnRH stimulates steroidogenesis in octopus testes and ovaries (48). As with other neuro-endocrine systems, the study of steroid hormones and steroid hormone receptors in lamprey might offer the opportunity of an insight into the origins of this regulatory module characteristic to vertebrates.

Steroidogenesis in lamprey is under the control of hypothalamic GnRH either directly, in experimental conditions or mediated by pituitary factors: surgical removal of hypophysis led to a drop in steroid plasma levels in female lampreys (49). GnRH-I and -III are equally potent in inducing secretion of estradiol after systemic administration in both adult males and females (50). Lamprey GnRH-I, -II as well as lGnRH-III stimulate *in vitro* release of estradiol from isolated lamprey gonads which demonstrates a direct action of GnRH on the reproductive organs *in vitro*. This effect strongly increases in co-culture systems where the gonads are exposed to GnRH treatment in the presence of pituitary tissue which convincingly supports the hypothesis of a hypophyseal gonadotropin-like factor with a GnRH-dependent release and having a steroidogenic effect on the gonads (24).

In lamprey, maturation of the gonads and gametogenesis are unique events in the life of the animal, initiated at the end of their parasitic phase and then progressing during the upstream migration of the animals to final ovulation and spermiation. Measurement of the plasma levels of classical sex steroids estradiol, progesterone, and testosterone in male and female lampreys during their final spawning phase revealed the presence of all three hormones albeit the testosterone was detected only at very low levels (51). Highest concentration of hormone was found for estradiol. The concentrations of steroids in the plasma measured in upstream migrating sea lamprey vary within a wide range of concentrations for all hormones but a number of significant trends can be identified in values obtained from observational experiments combined with hypothesis driven manipulative experiments [reviewed in Ref. (52)]: (a) in most cases all sex steroids are present at detectable, albeit in some cases very low levels, (b) there is a general trend for males to have higher levels of steroid hormones in the plasma, (c) there is a general trend for a decrease in the hormone levels from higher values during the migration to lower concentrations at spawning, (d) estradiol appears to be the most sensitive various conditions during reproductive phase of the animal, and (e) classical steroid hormones in lamprey respond to systemic administration of GnRH. Different types of stereotypical behaviors exhibited by the animals during spawning were correlated with significant changes in the levels of estradiol detected in both males and females (53).

While the data available for the relationship between plasma testosterone level and reproductive physiology of lamprey provides little support for a role of this steroid in lamprey, some evidence has been accumulated in respect to the role of a metabolic precursor of testosterone, the androstenedione. A cytosolic extract of lamprey testes was showed to bind androstenedione with an affinity about five times higher than its binding affinity for testosterone. Given this high affinity it has been proposed that low levels of androstenedione in the plasma after treatment with lamprey GnRH can be explained by its sequestering in the testis in a form bound to this putative receptor. Administration of androstenedione to pre-spermiating males accelerated the rate of maturation of their gonads (52, 54). The observation that injection of reproductively mature lamprey with GnRH or pituitary extract induces a significant increase in plasma levels of 15-hydroxylated forms of progesterone and testosterone led to the hypothesis that these steroid derivatives might act as unconventional sex hormones in lamprey (55, 56).

Actions of sex steroids at a central level in vertebrates can be grouped in three types of effects: (1) negative feedback – neuro-endocrine homeostatic mechanism of control of gonadal steroidogenesis, (2) positive feedback – has a physiologic role during the LH surge and ovulation in the estrous cycle of mammals, and (3) behavioral neuromodulators upon binding to a multitude of steroid sensitive brain areas. In female vertebrates gonadal steroid hormones regulate activity on the HPG axis via both positive and negative central feed-back mechanisms, these mechanisms being the most prominent and most studied during the estrous cycle of mammals (42).

The neuromodulatory role of steroids depends on the spatial and temporal pattern of expression of different types of steroid receptors in the brain tissue. First evidence of steroid feed-back action in lamprey was provided by estrogen brain tissue autoradiography experiments (57). Early experiments on binding affinity between estradiol and protein fractions of nuclear and cytosolic extracts from lamprey testes suggested presence of ERs in the lamprey gonadal tissue as well (58). Extensive PCR screening with degenerate primers of total RNA samples extracted from lamprey liver led to identification of three steroid hormone receptor paralogs (59). Molecular phylogeny analysis of the corresponding sequences suggested that they are orthologs of the three main groups of Gnathostome steroid hormone receptors: lamprey ER is an ortholog of estradiol receptors α and β, the second receptor (termed lamprey progesterone receptor, PR) is an ortholog of Gnathostome PR, and the third (lamprey corticoid receptor, CR) is similar with mineralocorticoid and glucocorticoid receptors. No androgen receptor sequence was obtained using this approach. This is consistent with a model of nuclear steroid hormone evolution where the testosterone selective receptors are a Gnathostome acquisition resulted from a duplication and functional divergence of the PR that took place after the split of the Cyclostome lineage (59).

The only available data on localization of the lamprey steroid hormone receptors in the brain was obtained by *in situ* hybridization by Sower and Baron (60) and indicate the presence of the only ER isoform identified in lamprey in the telencephalon (the periventricular area of the olfactory bulb), in the middle hypothalamic, and the habenular regions of the diencephalon as well as in the medial midbrain. The precise homology relationship of the ER sensitive area in the lamprey brain as indicated by these results with the components of the steroid hormone sensitive network of brain structure known to be involved in regulation of social behavior in vertebrates still needs to be established. However, their position is consistent with data obtained in teleosts for steroid hormone receptor expression in the brain (possibly the lateral septum anterior and ventromedial hypothalamus and periaqueductal gray) (61). Presence of steroid hormone receptor in the hindbrain may indicate sensitivity of local locomotor pattern generators to steroid input. Interestingly however, the estradiol receptor seems to be lacking in the preoptic area of the lamprey hypothalamus which is one of the most important regions for the control of the reproductive behavior in vertebrates (60). This raises the question whether there are not other types of steroid receptors including another ER expressed in this area. Androstenedione and hydroxylated steroids [reviewed in Ref. (52)] are non-classical steroid molecules candidates for a role, particularly androgenic, in reproductive physiology of lamprey but very little is known to date in respect to their activity in the central nervous system of this animal.

## Pheromonal Communication in Lamprey

Pheromones are intraspecific chemical communication devices. Chemicals released in the environment by an individual are capable of inducing specific effects in members of the same species via unlearned, genetically encoded mechanisms (62, 63). Pheromones usually stimulate olfactory sensory structures of conspecifics and convey their information via specialized or unspecific neural pathways and induce either behavioral or physiological effects. In the first case the effect is usually fast and triggers immediate social interaction responses related to aggression, mating, parental care, aggregation, etc., in which case they are termed *releaser* pheromones. In the second instance the effect is slower and more profound by inducing changes in the internal status of the recipient by activation of specific neuro-endocrine systems, in which case they are called *primer* pheromones. Releaser and primer pheromone systems have been described in many species of invertebrates, particularly in insects as well as in vertebrates. In the latter, all main groups of Gnathostomes with the exception of birds show some form of pheromonal communication. An important evolutionary event in this type of intraspecific communication in Gnathostomes was the development of specialized olfactory sensory structures and of the corresponding accessory neural olfactory pathways after divergence of Tetrapods (63). The most studied pheromone sensing structure is the vomeronasal organ, present in all Tetrapod branches with the exception of birds but absent in fish. This organ is also absent in apes although some vestigial, pseudogene, or inactive vomeronasal receptors have been identified for example in human genome.

Many years of observation and laboratory investigation of the factors that affect sea lamprey reproductive behavior both prior and during their upstream migration as well as during their final spawning phase have clearly indicated that olfactory cues play a central role in regulation of all these events (15). This acquired knowledge helped in the development of more efficient methods of control for lamprey population in the Great Lakes (10). The first conspecific chemical signal that directs the reproductive behavior in lamprey is a rare case when this type of communication take place between different stages of a vertebrate life cycle: it was shown that pheromonal cues released by the population of larvae in a stream not only will increase the eligibility of that stream but migrating animals will actively avoid streams with a low population of larvae (64). The chemicals responsible for this effect were found to be the larval 5-α bile acids isolated from the larval gall bladders (16, 65, 66). Petromyzonamine disulfate (PADS) is the most potent component, inducing the highest behavioral response while petromyzosterol disulfate and petromyzonal sulfate seem to be less important (64). A second type of pheromone described in sea lamprey was shown to guide the male-female interaction prior and during spawning. The bile acid 7α,12α,24-trihydroxy-5α-cholan-3-one 24-sulfate (3kPZS) was isolated from water conditioned with spermiating males. The compound was demonstrated in the liver of migrating animals and was shown to be released most likely from the gills of the animal and to specifically attract ovulating females in behavioral tests (67–69). More recently, the electro-olfactogram of another polyhydroxy steroid isolated from spermiating male washings, petromyzosterol was shown to stimulate the female olfactory system albeit in a much lower extent than 3kPZS (70).

Does lamprey have an accessory olfactory system? The vomeronasal system is a chemical sensory system that is anatomically, functionally, and neuroanatomically distinct from the main olfactory system in Tetrapods (63). This organ is considered absent from Actinopterygians but at the cellular level, the fish olfactory epithelium is a mosaic of olfactory sensory neuron types and some of these types share characteristics with the olfactory neurons present in the vomeronasal organ. This suggests that the fish main olfactory epithelium may accomplish the same functions related to pheromone detection that in later evolved lineages were delegated to a distinct organ. Investigation of the complement of olfactory sensory neurons in lamprey revealed the same kind of polymorphism as found in teleosts which may indicate that the olfactory epithelium in lamprey may have “normal” as well as pheromonal detection roles (71).

The distinction between the normal and pheromonal olfactory input however pertains to configuration of the neural pathways that connect the peripheral sensory system to the integrative centers of the brain, in particular the GnRH system and the neural structures that direct the reproductive behavior of the animal. Recently a direct neural pathway between olfactory input and the locomotor command centers in the reticulospinal formation of the hindbrain was described in sea lamprey using a combined electrophysiological, calcium imaging, and tract tracing approach (72). This outlines the neural substrate for conversion of olfactory signals (either feeding or reproduction related) into a motor behavioral output. Understanding the mechanism by which the locomotor output reflects internal needs related to reproduction will require identification of the nodes that connect this pathway with the steroid sensitive structures and with the GnRH system.

In Gnathostomes there are two sides of the relationships between olfactory input and GnRH system, related to the hypophysiotropic versus non-hypophysiotropic (neuromodulatory) roles of GnRH: (1) in the first case the olfactory input with reproductive relevance modulates the secretion of the GnRH from the preoptic area of the hypothalamus and consequently the activity on the HPG axis and (2) non-hypophysiotropic GnRH regulates the receptivity to olfactory stimuli via centrifugal projections to the peripheral olfactory structures:

(1) The first type of interaction can be described as a pheromone priming effect on GnRH secretion. Recent evidence suggests that chemosensory input induce upregulation of GnRH expression in the lamprey brain followed by the downstream activation of HPG pathway and increase in plasma steroid level (73). The pathways that connect olfactory input to activation of GnRH secretion in the preoptic area are not known.
(2) The second instance of the relationship between GnRH and olfaction is represented by the neuromodulatory (non-hypophysiotropic) effect of GnRH on olfactory sensory pathways. In most vertebrates, this role is studied in connection with the GnRH system attached to *nervus terminalis* and olfactory sensory epithelium: the neurons of the *nervus terminalis* ganglia were shown to express GnRH and send projections peripherally to the olfactory epithelium neurons that express GnRH receptors (74). Centrifugal neuromodulatory input to GnRH neurons of the *nervus terminalis* convey integrative signals to the chemosensory neurons in the nasal cavity thus adjusting the olfactory sensitivity to reproductive needs. In lamprey the existing experimental data does not support a neuromodulatory role of extra-hypothalamic GnRH on olfactory pathways similar with Gnathostomes. The decades long dispute about the existence of a *nervus terminalis* in this animal has been settled with a consensus that lamprey lacks this neural structure (75). In the only instance where lGnRH-II transcripts were reported in the olfactory bulb the *in situ* results could not be reproduced by immunohistochemistry (24). More recently receptor transcripts for lamprey GnRH-R 3 were detected in the olfactory bulb of females but not in males (37).

In conclusion, the influence of pheromonal signals on lamprey behavior has been well established at different stages of its reproductive cycle and they also seem to induce priming effects on GnRH. The neural pathway that mediate these actions are largely unknown in both of cases. Moreover, no evidence of a non-hypophysiotropic, neuromodulatory role of lamprey GnRH on olfaction has been found to date.

## Effect of Photoperiod and Temperature on Lamprey Reproduction

The key players in photoperiod neuro-endocrine regulation in vertebrates are the pineal gland and its hormone, melatonin. In adult mammals the pineal does not directly respond to light stimuli, this information if received at the central level from the retina via a specialized retinofugal pathways, the retinohypothalamic tract [see for example Ref. (76)]. This centripetal tract conveys photoperiod information to the supra-chiasmatic nucleus (SCN) of the hypothalamus which is the master circadian rhythm generator in the vertebrates. SCN processes the ambient light information and controls the release of melatonin from the pineal gland via noradrenergic stimulation at the end of a long multisynaptic path that includes nuclei from hypothalamus, spinal cord, and superior cervical ganglion. Melatonin diffuses into the bloodstream and is distributed back to the brain where it regulates photoperiod linked neural and neuro-endocrine mechanisms via melatonin sensitive nuclei distributed all over the brain. GnRH neurons are not directly affected by melatonin but this effect is mediated by other neuromodulators which more recently have been identified with the hypothalamic kisspeptin (77) and GnIH (2) in birds, or its ortholog RFamide related peptide (RFRP) in mammals (78, 79).

In lamprey both retinofugal projections to hypothalamus and optic tectum as well as retinopetal FMRF-amide immunoreactive projections connecting the visual centers in the brain with the eye have been described (80). Similarly with a number of fish and reptile species the lamprey pineal is directly sensitive to light signals (81), and therefore it is potentially capable of directly converting light signals into a melatonin secretion pattern. This gland appears like a vesicle connected to the posterior commissure through a long stalk and is part of the pineal complex which includes also the parapineal organ located ventral to the pineal (82). In addition to retinal and pineal photosensitive structures, it must also be noted that in lamprey there is evidence of encephalic or “deep-brain” photoreceptors that may convey light information directly to the specific brain systems which they are attached to (83).

Lamprey behavior during the last, reproductive phase of their life cycle is sensitive to the dark/light cycle. Observations of animals during their migration and spawning suggest they swim upstream mostly at night while spawning takes place during the day (18). As described above water temperature is an important environmental factor as well, the animals cease their upstream migration when the temperatures falls under certain species and location specific values. Moreover, it was shown that temperature is correlated with plasma estradiol levels (50) as well as with a sexually dimorphic profile of the brain levels of GnRH-I, -II, and -III (84). Walaszczyk et al. (85) have observed the locomotor rhythms in female lampreys in quasi-natural laboratory conditions and the effect of internal (sexual maturation, pre-ovulated vs. ovulated) and external (pheromone exposure) on these rhythms. The dominant pattern follows the photoperiod, with bursts of activity which are at their highest at the beginning of the night followed by a slow decrease toward the hours of the morning and into the day. Sexual maturation and presence of pheromones each has an effect on the relative intensity of locomotor activity without changing the diel pattern. Intersection of these two factors alters this pattern by inducing a new peak of activity during the day concomitantly with a reduction in activity at the beginning of the night in ovulated females exposed to sex pheromones. The temperature during the experiments was relatively constant around 17°C.

Measurement of the melatonin release from *in vitro* cultured lamprey pineal glands under different light and temperature regimes has shown that under an alternating light:dark regime at 20°C the melatonin secretion was completely inhibited during the light cycle and peaked in the dark (86, 87). The amplitude of melatonin rhythms are dependent on the ambient temperature, with day/night differences tending to level off as the temperature drops under 10°C. Both light and low temperatures are well known factors controlling the behavior of the migrating lampreys. *In vitro* sensitivity of the pineal to temperature suggests that melatonin might be also implicated in the neuro-endocrine response to temperature regime during migration. Lamprey migration is the sum of a number of distinct behaviors that are sensitive to environmental conditions but driven by genetically encoded responses to social (pheromonal) and internal hormonal stimuli.

Photoperiod affects the neuro-endocrine reproductive axis at different levels [reviewed for example in Ref. (88)]. Interaction between photoperiod and reproduction may occur at the interface between melatonin sensitive and steroid sensitive brain areas. These interactions may propagate to either behavioral changes as described above or to alterations of neuro-endocrine transduction to GnRH secretion in the ventral hypothalamus. There is limited data describing the diel profiles of steroid and GnRH in lamprey. Information regarding the brain profiles of steroid receptor expression and of melatonin receptor expression is also limited. Steroid receptor expression in lamprey brain was investigated in Ref. (84). Intense autoradiography signals with [2-^125^I]-melatonin was detected in the lamprey optic tectum and in the parvocellular and magnocellular cells of the preoptic nucleus (89). Co-localization of melatonin receptor/binding sites and GnRH secretion in the POA area of the lamprey hypothalamus opens the possibility of a direct effect of melatonin on GnRH neurons. But this needs more investigations before becoming a hypothesis. Another instance of a potential direct interaction with the GnRH system is the expression of the lamprey GnRH receptors in the pineal gland (37). GnRH receptor was also detected in the eyes of the animal (36). However interesting they are, the significance of these findings is difficult to assess at this moment due to lack of a clear mechanism by which GnRH ligands may reach these extra-pituitary receptors.

In summary, light and temperature are important environmental factors that directly impact the behavior of the animals during their reproductive phase, possibly through mechanisms that involve the pineal and its melatonin secretion. The effect of these factors are sensitive to the combined influence of gonadal maturation stage and the presence of olfactory signals. As in many other cases in lamprey, there is only a limited understanding of how these interrelations are determined at the central level possibly through interplay between the melatonin and steroid sensitive brain pathways.

## Concluding Remarks

Our goal was not to give an exhaustive presentation of the research in lamprey reproduction as there is not enough room for that in the space of a short review. Nor are we attempting to provide a comprehensive model for the control of reproduction in lamprey as this would be condemned to be highly speculative at this time given the rather fragmentary information available on molecular mechanisms that drive reproduction in this animal. Instead we adopted here a perspective on lamprey reproduction as an integrated system that in its most basic organization is an input/output system reflective to a certain extent of the primordial neuro-endocrine system that governed reproduction at the beginning of the vertebrate radiation. We briefly surveyed some of the most important channels through which the organism collects information about the internal and external media. This information is processed to generate an output represented by a sequence of stereotyped behaviors and stages of maturation of reproductive organs. The data accumulated to date suggests that lamprey share many of the neuro-endocrine characteristics of Gnathostomes like the existence of a HPG axis, the role of the sex steroid hormones, and the sensitivity of reproduction to light and temperature. These processes are coordinated at the central level by mechanisms that in lamprey are likely genetically encoded and inscribed into the morphological and chemical makeup of the brain during the embryonic and metamorphic development. Aside from the central role of the GnRH however, much less is understood from reproductive neurobiology of lamprey and of agnathans in general. There is an important body of information available on chemical neuroanatomy of lamprey brain, collected more recently through the efforts of research groups like Pombal in Spain (90) or Grillner in Sweden (91) among others. These efforts contributed to a deeper understanding of the evolutionary developmental origins of the vertebrate brain and also they allowed the lamprey locomotor system to become one of the best understood in vertebrates. Integration of the approaches used in these fields of interest within a framework more interested in reproductive neuroendocrinology may help unravel the interactions between peripheral and central levels of control of reproduction in agnathans and consequently of the most basic reproductive mechanisms in all vertebrates.

## Conflict of Interest Statement

The authors declare that the research was conducted in the absence of any commercial or financial relationships that could be construed as a potential conflict of interest.
